# Aging-Related Systemic Manifestations in COPD Patients and Cigarette Smokers

**DOI:** 10.1371/journal.pone.0121539

**Published:** 2015-03-18

**Authors:** Laurent Boyer, Christos Chouaïd, Sylvie Bastuji-Garin, Elisabeth Marcos, Laurent Margarit, Philippe Le Corvoisier, Laetitia Vervoitte, Leila Hamidou, Lamia Frih, Etienne Audureau, Ala Covali-Noroc, Pascal Andujar, Zakaria Saakashvili, Anne Lino, Bijan Ghaleh, Sophie Hue, Geneviève Derumeaux, Bruno Housset, Jean-Luc Dubois-Randé, Jorge Boczkowski, Bernard Maitre, Serge Adnot

**Affiliations:** 1 APHP, Hôpital Henri Mondor, DHU-ATVB, Département de Physiologie-Explorations Fonctionnelles, F-94010, Créteil, France; 2 INSERM U955 and Université Paris Est (UPEC), UMR U955, Faculté de médecine, F-94010, Créteil, France; 3 Centre Hospitalier Intercommunal, DHU-ATVB, Département de Pneumologie et Pathologie Professionnelle, F-94000, Créteil, France; 4 APHP, Hôpital Henri Mondor, Département de Santé Publique, F-94010, Créteil, France; 5 Université Paris Est (UPEC), Faculté de médecine, LIC, EA4393, F-94010, Créteil, France; 6 INSERM, Centre d’Investigation Clinique 1430, AP-HP, Hôpital Henri Mondor, F-94010, Créteil, France; 7 APHP, Hôpital Henri-Mondor, Département d’Addictologie, F-94010, Créteil, France; 8 APHP, Hôpital Henri Mondor, Plateforme de Ressources Biologiques, F-94010, Créteil, France; 9 APHP, Hôpital Henri Mondor, Département d’Immunologie Biologique, F-94010, Créteil, France; 10 APHP, Hôpital Henri Mondor, DHU-ATVB, Département de Cardiologie, F-94010, Créteil, France; Helmholtz Zentrum München, GERMANY

## Abstract

**Rationale:**

Chronic obstructive pulmonary disease (COPD) is often associated with age-related systemic abnormalities that adversely affect the prognosis. Whether these manifestations are linked to the lung alterations or are independent complications of smoking remains unclear.

**Objectives:**

To look for aging-related systemic manifestations and telomere shortening in COPD patients and smokers with minor lung destruction responsible for a decline in the diffusing capacity for carbon monoxide (DL_CO_) corrected for alveolar volume (K_CO_).

**Methods:**

Cross-sectional study in 301 individuals (100 with COPD, 100 smokers without COPD, and 101 nonsmokers without COPD).

**Measurements and Main Results:**

Compared to control smokers, patients with COPD had higher aortic pulse-wave velocity (PWV), lower bone mineral density (BMD) and appendicular skeletal muscle mass index (ASMMI), and shorter telomere length (TL). Insulin resistance (HOMA-IR) and glomerular filtration rate (GFR) were similar between control smokers and COPD patients. Smokers did not differ from nonsmokers for any of these parameters. However, smokers with normal spirometry but low K_CO_ had lower ASMMI values compared to those with normal K_CO_. Moreover, female smokers with low K_CO_, had lower BMD and shorter TL compared to those with normal K_CO_.

**Conclusions:**

Aging-related abnormalities in patients with COPD are also found in smokers with minor lung dysfunction manifesting as a K_CO_ decrease. Decreased K_CO_ might be useful, particularly among women, for identifying smokers at high risk for aging-related systemic manifestations and telomere shortening.

## Introduction

Chronic obstructive pulmonary disease (COPD) is associated with systemic manifestations including atherosclerosis, weight loss, osteoporosis, muscle atrophy and weakness, kidney dysfunction, and diabetes.[1–7] The mechanisms linking this systemic component to the lung disease remain unclear. Most of the studies addressing this issue found no association between systemic manifestations and degree of airflow limitation.[2] Associations with the severity of lung emphysema or of systemic inflammation have been reported but remain poorly understood.[5, 7–10] Improved understanding of the pathogenesis of systemic alterations in COPD may result in better preventive and therapeutic strategies.

COPD is an age-related condition, and accumulating evidence suggests a relationship with a global process of accelerated aging. Patients with COPD exhibit telomere shortening in circulating leukocytes compared to smokers without COPD [11, 12]. New data support an association between COPD and exaggerated lung-cell senescence that may contribute to the pathogenesis of the disease.[13–16]. Thus, one current hypothesis in COPD ascribes the systemic manifestations to a global aging process. Another possibility is that smoking, which is the most common cause of COPD, is also responsible for the systemic manifestations of the disease, independently from the lung function alterations [17]. We reasoned that comparing patients with COPD to smokers and nonsmokers might shed light on the role for lung alterations in the systemic aging-related manifestations. Moreover, we hypothesized that, in smokers without COPD, systemic manifestations may occur more frequently in individuals with than without lung dysfunction. One way to assess lung function in smokers is DL_CO_ measurement, as a decrease DL_CO_ has been proven sensitive for detecting lung dysfunction, even in patients without emphysematous lesions by computed tomography (CT) [18]. Analyses for data from smokers with lung dysfunction are not confounded by COPD-related factors such as pharmacological treatments, physical inactivity, exercise limitation, and gas exchange alterations. Furthermore, there are differences in the aging process between men and women [12, 19]. We therefore investigated three groups of individuals, patients with COPD, smokers without COPD (control smokers), and nonsmokers, taking into account cigarette-smoke exposure and gender.

## Materials and Methods

In this cross-sectional study, we enrolled 301 participants including 100 with COPD, 100 smokers without COPD, and 101 nonsmokers, recruited at the Henri-Mondor Teaching Hospital, Créteil, France, between January 2009 and September 2012. The study was approved by the institutional review board of the Henri-Mondor Teaching Hospital (CPP, # 09–027). All participants provided written informed consent before inclusion.

### Study population

Patients with clinically stable COPD were recruited prospectively at the pulmonology outpatient clinic and control smokers at the smoking-cessation clinic and at the clinical investigations center of the Henri-Mondor Teaching Hospital. Nonsmokers were healthy volunteers recruited from the general population by the clinical investigations center of the same hospital and evaluated clinically before study inclusion. Exclusion criteria were known chronic heart failure, malignancy, and inflammatory or metabolic conditions.

Each participant underwent spirometry, plethysmography, and DL_CO_ measurement according to ATS/ERS consensus guidelines.[20] DL_CO_ was measured using the single breath method. K_CO_, which is DL_CO_ corrected for alveolar volume, was used for the analysis. DL_CO_ and K_CO_ were corrected for hemoglobin.

Control smokers and nonsmokers were required to have FEV_1_/FVC greater than 70%. Smokers without COPD were classified as having reduced K_CO_ (<80%) or normal K_CO_ (≥80%).

### Aging-related systemic manifestations

Arterial stiffness (aortic pulse-wave velocity, PWV) was measured as the carotid-femoral pulse-wave velocity using the Complior Analyse device (Alam Medical, Vincennes, France) and was available in 294/301 subjects. Bone mineral density (BMD) at the hip (femoral neck) and lumbar spine was determined using dual-energy X-ray absorptiometry (Lunar iDXA, GE Healthcare, UK). Complete dataset was obtained in 295/301 subjects. BMD is reported as the absolute value (g/cm^2^). T-scores were computed to classify participants as having normal BMD or osteoporosis (defined as T-score <-2.5 at either site). Appendicular skeletal muscle mass (ASMM) was measured as the fat-free soft-tissue masses of the arms and legs divided by height squared [21] and ASMM index (ASMMI) was then computed as ASMM divided by height squared. The cutoff for defining sarcopenia was two standard deviations below the mean sex-specific ASMMI values in the Rosetta Study of young adults (5.45 for females and 7.26 for males), as proposed by Baumgartner et al. [21]. Pinch and grip strengths were assessed using a standard handgrip dynamometer and pinch gauge (Baseline Evaluation Instruments, NY, USA), insulin resistance by calculating HOMA-IR (insulin·glucose)/22.5), and renal function by estimating the glomerular filtration rate (eGFR) using the Cockcroft-Gault formula.

### Blood tests

Levels of 27 serum chemokines and growth factors were quantified using a bead-based cytometric immunoassay (Bio-Rad Laboratories, Hercules, CA). Among the 27 chemokines and growth factors, we focused on 12 for subsequent analysis in patients with COPD, control smokers, and nonsmokers (IL-1β, IL-1Ra, IL-6, IL-8, IFN-γ, IP-10, MCP-1, MIP-1α, MIP-1β, RANTES, TNF-α, eotaxin). Measurement of telomere length in circulating leucocytes was assessed using the real-time quantitative polymerase chain reaction (RT-qPCR).[12] Telomere length measurement was obtained in 89/100 COPD patients, 90/100 control smokers and 94/101 non smokers.

### Statistical analysis

Qualitative variables are reported as numbers and percentages, and quantitative variables as median, interquartile range, IQR.

Pulmonary function parameters, aging-related systemic manifestations, leukocyte counts, and plasma cytokine levels were compared across the three groups (COPD patients, control smokers, and nonsmokers) using the Chi2, Fisher exact, or Kruskal-Wallis test, as appropriate. When differences were significant, COPD patients were compared with control smokers, and control smokers with nonsmokers. After looking for a first-order interaction, to take into account potential confounding the analyses were also adjusted for gender and age (and pack-year value for the comparison of COPD patients to control smokers) by fitting logistic models for qualitative variables and nonparametric quantile regression models for quantitative variables. These models provide estimates of the median values of the dependent variable in each group conditional on the value of confounders [22]. Bootstrap resampling with 100 replications was performed to correct for potential heteroskedastic errors.

To assess whether minor lung destruction was associated with extrapulmonary manifestations of aging, we compared aging-related systemic manifestations, leukocytes, and cytokines between control smokers with normal vs. low K_CO_, using similar analyses. BMD and telomere length were performed separately in men and women because gender modified the relationships between these factors and K_CO_ (significant interaction).

All tests were two-tailed. *P* values ≤0.05 were considered significant. Data were analyzed using Stata statistical software (StataCorp 12.1, College Station, TX).

## Results

### Clinical characteristics of the study population

Age, body mass index, and mean systemic arterial pressure did not differ across the three groups but COPD patients had a higher pack-year value compared to control smokers (Table 1). All the COPD patients were smokers, the percentage of current smokers was 46% among patients with COPD and 71% among control smokers (Table 1). Both DL_CO_ and K_CO_ were lower in COPD patients than in control smokers and in control smokers than in nonsmokers. The groups did not differ regarding treated diabetes or medical history. Inhaled steroids were used by 45 (45%) patients with COPD (S1 Table). Patients with COPD exhibited different levels of airflow limitation: GOLD stage was I in 8% of patients, II in 49%, III in 32%, and IV in 11%.

**Table 1:** Characteristics of the study patients.

	Nonsmokers (n = 101)	Smokers without COPD (n = 100)	Patients with COPD (n = 100)	*P* value †
Age, years	59.5 [53.3–63.6]	59.6 [53.6–64.1]	60.6 [56.7–65.9]	0.11
Females/males, n	36/65	41/59	28/72	0.15
Pack-years		30 [24–41]	41.5 [32–62]	<0.001
Current smokers, n (%)		71 (71)	46 (46)	<0.001
BMI, Kg/m^2^	25.6 [23.2–28.0]	25.5 [22.8–27.8]	25.7 [22.4–29.0]	0.91
MAP, mmHg	92 [87–98]	93.3 [87–98]	93.8 [87–100]	0.58
**Pulmonary function parameters**				
FEV_1_, L	3.2 [2.5–3.8]	2.9 [2.4–3.5]	1.4 [1.1–2.0]	0.000
FEV1, % predicted	109 [97–117]	101 [90–112]	55 [38–69]	0.000
FVC, L	3.9 [3.1–4.6]	3.7 [3.0–4.5]	3.0 [2.3–3.6]	0.000
FVC, % predicted	101 [90–116]	100 [88–112]	78 [65–93]	0.000
FEV1/FVC	81 [78–85]	79 [76–83]	51 [42–64]	0.000
DL_CO_, % predicted	87 [77–97]	80 [69–88.5]	54 [44–73]	0.000
K_CO_, % predicted	92 [82–105]	83 [72–95]	67 [52–82]	0.000
SpO_2_, %	97.0 [96.4–97.7]	97.0 [96.4–97.5]	96.3 [95.2–97.0]	0.000
6-min walking distance, m	600 [540–636]	579 [510–627]	510 [450–570]	0.000

*Definition of abbreviations*: COPD, chronic obstructive pulmonary disease; % predicted, percentage of the predicted value; BMI, body mass index; MAP, mean arterial pressure; K_CO_, transfer factor coefficient of the lung for carbon monoxide; SpO_2_, oxygen saturation by pulse oximetry.

Data are median [interquartile range] unless stated otherwise.

†*P* value by Chi-square test, Fisher exact test, or nonparametric Kruskal-Wallis test, as appropriate, comparing the three populations (COPD patients, control smokers, and nonsmokers).

### Aging-related systemic manifestations, telomere length, and cytokine plasma levels in patients with COPD compared to control smokers and nonsmokers

**Fig 1:**
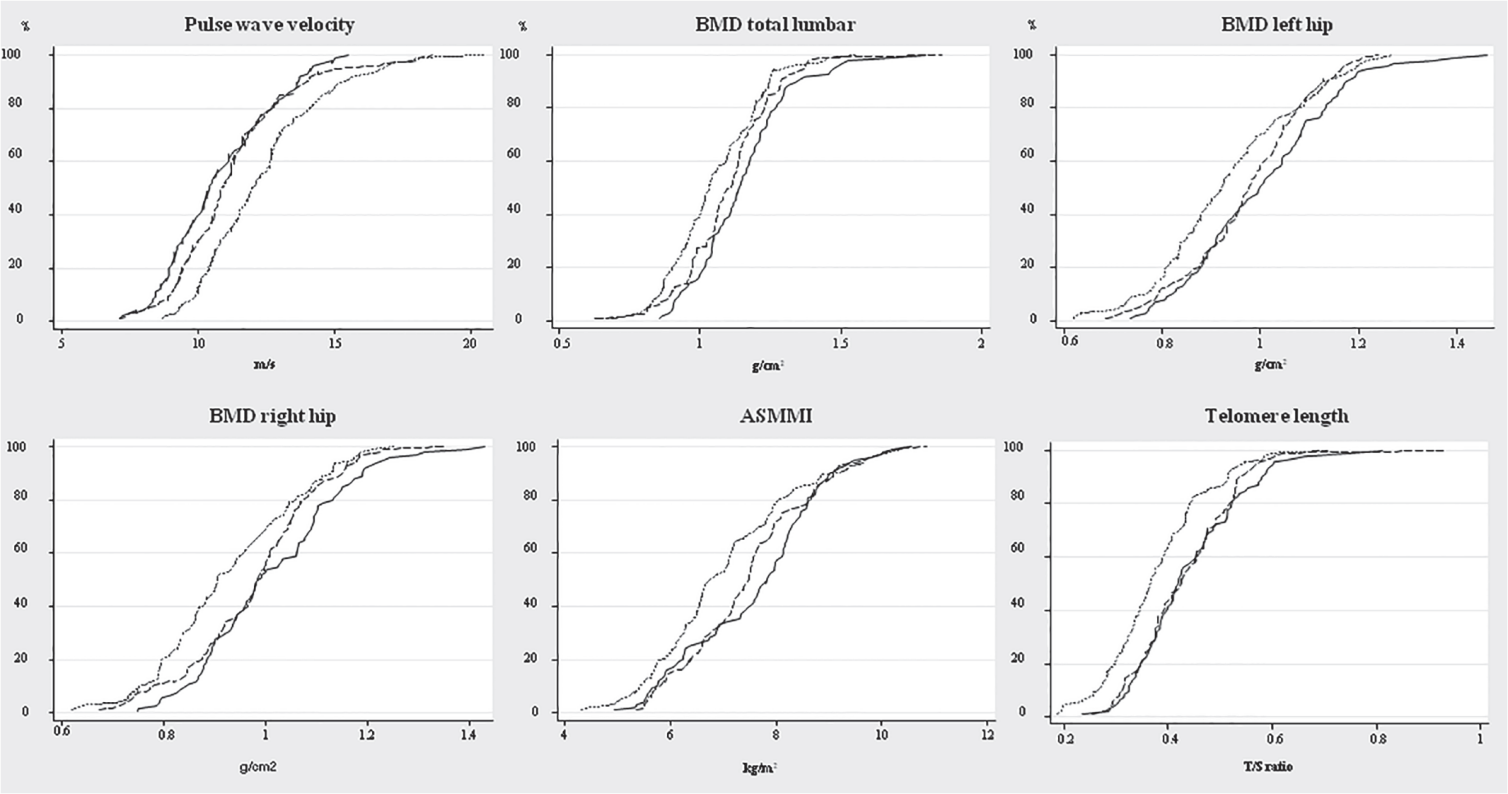
Plots of cumulative percentages of aging-related systemic manifestations and telomere length in nonsmokers (—), control smokers (without COPD, ---) and COPD patients (….).

The three groups differed significantly regarding PWV, lumbar-spine and hip BMD, ASMMI, and TL when they are compared globally; but not HOMA-IR, eGFR, or muscle function tests (Table 2, Fig. 1). Compared to control smokers, patients with COPD had higher PWV and lower lumbar-spine and hip BMD, ASMMI, and TL; even after adjustment for age, gender, and pack-year history. Median values were not affected by adjustments. Sarcopenia was found in 21% of patients with COPD compared to 2% of control smokers (*P*<0.001) and osteoporosis in 21% of patients with COPD compared to 12% of control smokers (*P* = 0.16). No significant differences in these variables were observed between control smokers and nonsmokers, before or after adjustment for age and gender (Table 2).

**Table 2:** Comparison of aging-related systemic manifestations and telomere length in patients with chronic obstructive pulmonary disease, smokers without COPD, and nonsmokers.

	Adjustment	Nonsmokers	Smokers without COPD	COPD patients	*P* value †	*P* value *	
						S vs NS	COPD vs S
**Pulse wave velocity, m/s**	none	10.4 [9.1–12,2]	10.9 [9.7–12.2]	12,1 [10.6–13.5]	**0.000**	0.14	**0.000**
	gender and age	10.6	11.1	12.0	**0.000**	0.23	**0.003**
	gender, age, and pack years	-	11.2	11.9	-	-	**0.03**
**BMD, total lumbar, g/cm^2^**	none	1.14 [1.04–1.24]	1.11 [0.99–1.19]	1.03 [0.93–1.18]	**0.000**	0.08	**0.02**
	gender and age	1.14	1.10	1.03	**0.009**	0.08	**0.05**
	gender, age, and pack years	-	1.09	1.04	-	-	0.09
**BMD, left hip, g/cm^2^**	none	1.00 [0.89–1.09]	0.98 [0.89–1.06]	0.92 [0.83–1.03]	**0.005**	0.27	**0.02**
	gender and age	1.00	0.99	0.91	**0.001**	0.55	**0.00**
	gender, age, and pack years	-	0.98	0.91	-	-	**0.00**
**BMD, right hip, g/cm^2^**	none	0.99 [0.90–1.10]	0.99 [0.89–1.06]	0.91 [0.83–1.04]	**0.000**	0.19	**0.01**
	gender and age	1,00	0.98	0.92	**0.02**	0.37	**0.03**
	gender, age, and pack years	-	0.98	0.93		-	**0.03**
**ASMMI, Kg/m^2^**	none	7.8 [6.4–8.5]	7.5 [6.6–8.1]	6.8 [6.10–7.9]	**0.004**	0.28	**0.01**
	gender and age	7.5	7.5	6.9	**0.03**	0.99	**0.03**
	gender, age, and pack years	-	7.4	6.9	-	-	**0.02**
**Sarcopenia, n (%) §**	none	3 (3)	2 (2)	26 (26)	**0.000**	0.70	**0.000**
	gender and age					-	**0.000**
	gender, age, and pack years					-	**0.000**
**Pinch test, Kg**	none	7 [5–9]	6 [5–8]	6 [5–8]	0.08	-	-
**Grip test, Kg**	none	38 [26–48]	38 [26–45]	36 [27–42]	0.39	-	-
**HOMA-IR**	none	1.5 [1.1–2.4]	2.0 [1.2–3.1]	2.2 [1.2–3.1]	0.10	-	-
**Glomerular flow rate, mL/min**	none	78.4 [68.9–89.6]	79.6 [69.7–91,8]	80.9 [69.8–94.6]	0.18	-	-
**Telomere length (T/S) ratio**	none	0.42 [0.36–0.51]	0.43 [0.36–0.50]	0.37 [0.31–0.4]	**0.000**	0.75	**0.000**
	gender and age	0.42	0.41	0.37	**0.006**	0.75	**0.05**
	gender, age, and pack years	-	0.43	0.37	-	-	**0.008**

*Definition of abbreviations*: COPD, chronic obstructive pulmonary disease; NS, nonsmokers; S, smokers; BMD, bone mineral density; ASMMI, appendicular skeletal muscle mass index; HOMA-IR, homeostatic model assessment of insulin resistance; T/S, ratio of telomere-repeat copy number over single-gene copy number

Glomerular flow rate was estimated using the Cockcroft-Gault formula.

Data are median [interquartile range] unless stated otherwise.

†*P* value by Fisher exact test, or nonparametric Kruskal-Wallis test, as appropriate, comparing the three populations (COPD patients, control smokers, and nonsmokers).

*P value of quantile regression models for adjusted analyses.

§ *P* value of logistic regression models for adjusted analyses.

Among the 12 chemokines analyzed, IL-6, IL-8, and MCP-1 differed significantly across the three groups (Table 3). However, after adjustment for age, gender, and pack-year of exposure, no statistically significant differences were found between COPD patients and control smokers or between control smokers and nonsmokers. Control smokers had higher peripheral leukocyte counts compared to nonsmokers, even after adjustment for age and gender (Table 3). No significant differences were observed between patients with COPD and control smokers.

**Table 3:** Comparison of leukocytes and plasma inflammatory markers in patients with chronic obstructive pulmonary disease, smokers without COPD, and nonsmokers.

	Adjustment	Nonsmokers	Smokers without COPD	COPD patients	*P* value †	*P* value *	
						S vs NS	COPD vs S
**Leukocyte, Giga/L**	none	5.4 [4.6–6.4]	6.5 [5.3–8.3]	7.3 [5.8–8.65]	**0.000**	**0.000**	0.13
	gender and age	5.5	6.6	7.1	**0.000**	**0.004**	0.19
	gender, age, and pack years	-	6.6	7.0	-	-	0.50
**IL-6, pg/mL**	none	14.7 [12.9–17.3]	15.7 [12.9–18.5]	16.5 [14.3–19.2]	**0.01**	0.16	0.15
	gender and age	14.7	15.5	16.4	**0.02**	0.19	0.17
	gender, age, and pack years	-	15.5	16.4	-	-	0.22
**IL-8, pg/mL**	none	43.5 [38.9–50.4]	47.0 [40.0–51.6]	48.8 [42.7–53.4]	**0.008**	0.17	0.11
	gender and age	43.2	46.2	48.4	**0.005**	0.07	0.16
	gender, age, and pack years	-	47.5	48.5	-	-	0.59
**MCP-1, pg/mL**	none	33.4 [27.6–42.2]	37.3 [27.8–50.2]	40.4 [28.1–56.8]	**0.02**	0.12	0.19
	gender and age	34.8	37.7	39.5	0.32	-	-
	gender, age, and pack years	-	36.5	39.1	-	-	0.56
**TNF-α, pg/mL**	none	68.6 [58.3–86.0]	67.8 [56.6–82.1]	68.8 [56.6–84.0]	0.83	-	-
**Eotaxin, pg/mL**	none	90.3	123.6	102.9	0.06	-	-

*Definition of abbreviations*: COPD, chronic obstructive pulmonary disease; IL, interleukin; MCP-1, monocyte chemotactic protein-1; TNF-α, tumor necrosis factor alpha

Data are median [interquartile range].

†*P* value by nonparametric Kruskal-Wallis test comparing the three populations (patients with COPD, control smokers, and nonsmokers).

*P value of quantile regression models for adjusted analyses.

### Aging-related systemic manifestations, telomere length, and plasma cytokine levels in control smokers with normal spirometry and low K_CO_ compared to control smokers with normal spirometry and normal K_CO_

Clinical characteristics were similar in these two subgroups but the percentage of women was significantly higher among control smokers with low K_CO_ (59% vs. 32% for normal K_CO_, *P* = 0.01). Pack-year exposure was similar in control smokers with low and normal K_CO_ (31 [25–43] vs. 30 [24–44], respectively, *P* = 0.77).

Because of a significant interaction (*P*<0.05) of gender with both BMD and TL, we analyzed these parameters separately in males and females. Low K_CO_ was associated with low hip and lumbar-spine BMD in females but not in males. TL was shorter in females with low K_CO_ compared to those with normal K_CO_ (Table 4). Low K_CO_ was associated with decreased ASSMI and muscle function (grip test) even after adjustment for age and gender but was not associated with PWV, HOMA-IR, or eGFR.

**Table 4:** Comparison of aging-related systemic manifestations and telomere length between smokers with low K_CO_ and those with normal K_CO_

	Univariate analysis			Adjusted for age and gender		
	Normal K_CO_ (n = 50)	K_CO_<80% (n = 39)	P*	Normal K_CO_ (n = 50)	K_CO_<80% (n = 39)	*P* value †
Pulse wave velocity, m/s	11.2 [10–12]	10.8 [9.7–12.2]	0.77			
BMD, total lumbar, g/cm^2^ **						
In females	1.19 [1.07–1.24]	0.98 [0.91–1.12]	**0.001**	1.18	1.02	**0.003**
In males	1.09 [1.05–1.29]	1.14 [1.06–1.16]	0.87	-	-	-
BMD, left hip, g/cm^2^ **				-	-	
In females	0.98 [0.93–1.12]	0.84 [0.76–0.94]	**0.002**	0.99	0.85	**0.007**
In males	0.99 [0.95–1.08]	1.02 [0.95–1.06]	0.92			
BMD, right hip, g/cm^2^ **						
In females	0.99 [0.95–1.08]	0.88 [0.74–0.92]	**0.01**	1.01	0.87	**0.000**
In males	1.01 [0.95–1.10]	1.01 [0.96–1.05]	0.71			
ASMMI, Kg/m^2^	7.7 [7.1–8.7]	6.9 [5.9–7.6]	**0.000**	7.6	7.00	**0.03**
Pinch test, Kg	6.5 [5–8.5]	6 [4–8]	0.13			
Grip test, Kg	39 [29–46]	32 [22–43]	**0.02**	40.3	32.0	**0.01**
HOMA-IR	2.18 [1.15–3.26]	1.94 [1.09–2.21]	0.29			
Glomerular flow rate, mL/min	84.9 [74.8–91.4]	74.5 [64.4–93.5]	0.12			
Telomere length (T/S) ratio**						
In females	0.52 [0.41–0.57]	0.40 [0.36–0.46]	**0.023**	0.50	0.40	**0.037**
In males	0.43 [0.4–0.5]	0.46 [0.4–0.5]	0.16			

*Definition of abbreviations*: K_CO_, transfer coefficient of the lung for carbon monoxide; BMD, bone mineral density; ASMMI, appendicular skeletal muscle mass index; HOMA-IR, homeostatic model assessment of insulin resistance; T/S, ratio of telomere-repeat copy number over single-gene copy number

Data are median [interquartile range].

* *P* value by nonparametric Kruskal-Wallis test

†*P* value by quantile regression models adjusted for age and gender unless stated otherwise

** Females and males were analyzed separately because of a significant interaction between gender and aging-related parameters.

Peripheral leukocyte counts and plasma cytokine levels did not differ between control smokers with low vs. normal K_CO_ (Table 5). Similar results were obtained when control smokers were classified based on DL_CO_ lower or greater than 80% (data not shown).

**Table 5:** Comparison of inflammatory mediators between smokers with low K_CO_ and those with normal K_CO_.

	Smokers without COPD		
	Normal KCO (n = 50)	KCO<80% (n = 39)	*P* value†
Leukocytes, Giga/L	6.3 [5.1–7.8]	6.9 [6,0–8.4]	0.19
IL-6, pg/mL	15.1 [12.9–18,5]	16.9 [13,3–18.5]	0.46
IL-8, pg/mL	44.7 [38,9–51.6]	47.5 [41,8–51.6]	0.30
MCP-1, pg/mL	35,8 [26.6–50,0]	38.7 [27.3–50,2]	0.62
TNF-α, pg/mL	66.5 [55.5–74.8]	70.0 [59.5–85.9]	0.35
Eotaxin, pg/mL	127 [92–185]	125 [61–171]	0.39

K_CO_, transfer factor coefficient of the lung for carbon monoxide; IL, interleukin; MCP-1, monocyte chemotactic protein-1; TNF-α, tumor necrosis factor alpha

Data are median [interquartile range].

† *P* value by nonparametric Kruskal-Wallis test

## Discussion

Our data show that patients with COPD exhibit aging-related systemic manifestations and telomere shortening. These two characteristics were not significantly different between control smokers and nonsmokers, indicating that both were chiefly ascribable to COPD. Among control smokers, systemic manifestations and telomere shortening were more common in the subgroup with normal spirometry but low K_CO_ than in those with normal K_CO_, particularly among women, supporting a link between lung dysfunction and aging-related systemic manifestations. Smokers susceptible to premature aging might therefore be identified based on a low K_CO_.

One major goal of this study was to evaluate whether age-related physiological and biological abnormalities occurred in patients with COPD compared to smokers without COPD and nonsmokers and whether these abnormalities were detectable in smokers with minor lung dysfunction defined as decreased CO diffusing capacity. Previous studies have been performed in patients with COPD or in smokers without COPD but did not assess lung function in the control smokers, leaving unclear whether the systemic manifestations were ascribable to smoking or to lung dysfunction [23]. Our patients with COPD had specific systemic manifestations that were not observed in control smokers, including increased arterial stiffness, bone and skeletal muscle loss, and TL shortening. Interestingly, patients with COPD did not differ from control smokers regarding insulin resistance or renal dysfunction, two other potential systemic manifestations of COPD. These data are consistent with recent studies showing that several systemic manifestations seen in COPD occur in combination [7]. A major finding from our study was that control smokers and nonsmokers did not differ regarding physiological and biological parameters. Thus, in the three groups, the physiological parameters and leukocyte TL changed in lockstep, with differences between COPD patients compared to control smokers and nonsmokers but no differences between control smokers and nonsmokers. These results establish that the systemic manifestations are chiefly ascribable to COPD and not to smoking.

Our results may appear to contradict previous studies documenting TL shortening in smokers.[23] Most of these studies did not involve lung function assessments and were therefore unable to evaluate TL independently from lung dysfunction. Here, we investigated smokers with normal spirometry and individualized a subgroup with minor lung dysfunction not detected by spirometry but responsible for a decrease in K_CO_. K_CO_ reflects functional alveolar-capillary bed integrity.[24]. Low K_CO_ was common among our control smokers without airflow obstruction, particularly among the women (57% compared to 25% in male smokers). The most striking results were obtained in female smokers with normal spirometry but low K_CO_, who exhibited decreased BMD and TL shortening. K_CO_ values lower than 80% were closely associated with systemic manifestations in female smokers and, to a lesser degree, in male smokers. K_CO_ measurement might therefore be valuable as a screening tool among smokers with normal spirometry, to identify individuals with early age-related systemic abnormalities. Low K_CO_ may reflect early lung destruction before COPD onset, as lower baseline K_CO_ is independently associated with worse symptoms and more rapid progression of emphysema and airflow limitation in heavy smokers [18, 25, 26]. However, further longitudinal studies are needed to determine whether smokers without airflow obstruction but with low K_CO_ are at high risk for developing accelerated aging, COPD, and systemic manifestations.

Differences between control smokers with low vs. normal K_CO_ are not confounded by COPD-related factors such as exercise limitation, steroid use, hypoxemia, decreased BMI, smoking history, and current versus former smoking. Control smokers with low vs. normal K_CO_ did not differ for age, BMI, smoking history, 6-minute walk test results, or O_2_ saturation; although they differed regarding systemic abnormalities. Moreover, most of our control smokers were current smokers. Thus, the differences found in our study between control smokers with low vs. normal K_CO_ are probably ascribable to the lung disease or the process causing it, and not to the above-listed COPD-related factors. K_CO_ is closely linked to the degree of lung emphysema in COPD patients. Low K_CO_ in smokers is believed to reflect incipient smoking-induced destruction of both the lung parenchyma and the lung vessels.[24] K_CO_ measurement can detect subtle lung dysfunction in patients with normal chest CT findings.[18] K_CO_ is associated with increased plasma levels of circulating endothelial microparticles released from activated or apoptotic endothelial cells, which reflect vascular damage that may constitute an early step antedating emphysema development.[24] A role for emphysema in COPD-related systemic manifestations is supported by studies showing correlations of emphysema with low BMD and increased arterial stiffness in smokers with and without COPD.[9, 10] Taken together, these findings support a link between early lung parenchyma destruction and age-related systemic manifestations of COPD.

Smokers without COPD are generally chosen as controls for comparisons with COPD patients, as they are believed to be free from lung disease [6, 17]. This approach does not consider the heterogeneity of smokers without COPD, some of whom have early minor lung alterations that may be associated with alterations in extrapulmonary organs. In a study of 60 ex-smokers with COPD detected by spirometry, 60 control ex-smokers without COPD, and 60 nonsmokers, the COPD patients and control smokers were not significantly different regarding the prevalence of risk factors and comorbidities, whereas these prevalences differed between control smokers and nonsmokers, challenging the view that comorbidities are ascribable to lung alterations alone and not to smoking [17]. However, K_CO_ was lower in the control smokers than in the nonsmokers (88% versus 98%). The comorbidity difference between control ex-smokers and nonsmokers may reflect the presence of minor lung dysfunction in many smokers without COPD.

A major issue is whether the pulmonary and systemic components of COPD result from a common pathogenic mechanism or whether the lung alterations drive the development of systemic senescence-related manifestations. Regarding the first hypothesis, accelerated aging, and not smoking, could be the main risk factor of developing both COPD and its systemic manifestations. Our observation in smokers with low KCO could suggest that accelerated aging could arrive early in the disease history. However, inclusion of COPD patients without a history of smoking or longitudinal studies evaluating aging manifestations in smokers are needed to answer this question. In support for the second hypothesis, we and others have shown that lung emphysema and COPD are associated with an accumulation of senescent cells in lung tissues.[13–16] These cells are still metabolically active and release many mediators that not only contribute to inflammation in COPD, but also can propagate the senescence process to neighboring cells in the lung and potentially to systemic organs in a paracrine manner.[27] Consistent with this mechanism, recent evidence indicates a role for circulating factors in aging-related manifestations.[28, 29] Candidate circulating factors consist of cytokines (e.g., IL-6 and IL-8), prostaglandins (e.g., PGE2), and chemokines (e.g., eotaxin) including serum interferon-gamma-inducible chemokines (e.g., IP-10).[15, 16, 28, 30] In the present study, some of these circulating factors were altered in patients with COPD and control smokers. However, IL-6, IL-8, IP-10, and eotaxin were not significantly different between control smokers with low vs. normal K_CO_. Of note is the previous finding of significant variations in endothelial microparticles in smokers with low K_CO_, indicating associated endothelial damage in the pulmonary vessels.[24] Further studies are therefore needed to identify circulating factors potentially involved in the aging process in patients with COPD or in smokers with early lung destruction manifesting only as low K_CO_.

## Supporting Information

S1 Table Medical history and pulmonary medications of the study patients. (DOC)
