# Supplementary material for: Aging-Related Systemic Manifestations in COPD Patients and Cigarette Smokers
Source: PLoS One. 2015 Mar 18;10(3):e0121539. doi: 10.1371/journal.pone.0121539 (PMC4364985; doi:10.1371/journal.pone.0121539)
Supplement: S1 Table — (DOC) [file pone.0121539.s001.doc]

**Table S1: Medical history and pulmonary medications**

|  | **Nonsmokers (n= 101)** | **Control smokers (n=100)** | **COPD patients (n=100)** | ***P* value †** |
| --- | --- | --- | --- | --- |
| **Medical history** |  |  |  |  |
| Acute myocardial infarction, n (%) | 2 (2) | 4 (4) | 6 (6) | 0·31 |
| Stroke, n (%) | 0 (0) | 1 (1) | 1 (1) | 0.59 |
| Peripheral arterial disease, n (%) | 0 (0) | 3 (3) | 7 (7) | 0·02 |
| Treated diabetes, n (%) | 4 (4) | 3 (3) | 8 (8) | 0.19 |
| Sleep apnea syndrome, n (%) | 9 (9) | 6 (6) | 6 (6) | 0.69 |
| **Medications, n (%)** |  |  |  |  |
| Inhaled anticholinergic |  |  | 47(47) | - |
| Inhaled long-acting β2-agonist |  |  | 58 (58) | - |
| Inhaled corticosteroids |  |  | 45 (45) | - |
| Oral corticosteroids |  |  | 4 (4) | - |

†*P* value by the Chi-square test comparing the three populations (patients with COPD, control smokers, and nonsmokers)
